# Case Report: Regenerative peptides for Inferior alveolar nerve injury: a narrative review with hypothesis-generating three clinical cases

**DOI:** 10.3389/froh.2026.1907109

**Published:** 2026-09-30

**Authors:** Scott Froum, Nathan Estrin, Gianna Pico, Mikhail Yakubov, Paras Ahmad, James LaValle

**Affiliations:** 1 Department of Periodontics, School of Dental Medicine, Stony Brook University, Stony Brook, New York, NY, United States; 2 School of Dental Medicine, Lake Erie College of Osteopathic Medicine, Bradenton, FL, United States; 3 Private Practitioner, New York, NY, United States; 4 NewYork-Presbyterian Hospital, New York, NY, United States; 5 College of Dental Medicine, Lincoln Memorial Hospital, Knoxville, Tennessee, United States; 6 American Academy of Anti-Aging Medicine (A4M), Boca Raton, FL, United States

**Keywords:** lingual nerve, mandibular nerve, peptides, regeneration, trigeminal nerve injuries

## Abstract

Inferior alveolar nerve (IAN) injury represents a substantial complication in oral and maxillofacial surgery, often leading to persistent neurosensory dysregulation with limited effective treatment options. This narrative review analyzes the biological rationale for regenerative peptides as potential adjuncts in peripheral nerve repair and presents three hypothesis-generating clinical cases managed using a multimodal approach. Experimental evidence suggests that peptides, such as BPC-157, thymosin *β*4 derivatives (TB-500), and ARA-290, may affect biological pathways implicated in peripheral nerve repair, such as inflammation regulation, cytoprotection, angiogenic signaling, extracellular matrix remodeling, Schwann cell biology, and axonal regeneration. Nonetheless, these processes have been demonstrated mainly in preclinical models and have not been directly established in patients with IAN injury. Three illustrative clinical cases treated with a multimodal method incorporating peptide agents, dexamethasone, and local anesthetic showed neurosensory recovery during follow-up. Nonetheless, owing to the uncontrolled nature of these observations, spontaneous recovery and other confounding factors cannot be excluded, and causality cannot be established. Consequently, a phase-based intervention framework is proposed, emphasizing a biologically informed phase-mediated management system. Despite promising biological rationale, peptide-based interventions remain investigational, with unresolved questions concerning safety, delivery, optimal dosing, and long-term outcomes. Rigorous, controlled clinical trials and standardized outcome measures are required to validate efficacy and define their role with contemporary management paradigms for IAN injury.

## 1 Introduction

Inferior alveolar nerve (IAN) injury remains one of the most consequential ramifications related to oral and maxillofacial surgical procedures, such as orthognathic surgery, bone grafting, endodontic interventions, third molar surgery, and dental implant placement (1, 2). While several injuries are transient and resolve spontaneously, some patients encounter consistent neurosensory dysfunction marked by anesthesia of the chin and lower lip, dysesthesia, hypoesthesia, or paresthesia (2, 3). These sensory dysfunctions can significantly undermine quality of life by influencing psychosocial well-being, facial expression, mastication, and speech (3). Despite progress in surgical planning, surgical approaches, and cone-beam computed tomography (CBCT), iatrogenic injury to the IAN remains a substantial clinical challenge, especially when recovery is prolonged or incomplete (1, 4).

**Table 1:** CARE case summary of three patients with inferior alveolar nerve injury managed with a multimodal peptide-based regenerative protocol.

Variable	Case 1	Case 2	Case 3
Age/Sex	38-year-old female	85-year-old patient	48-year-old female
Etiology of IAN injury	Implant placement at site #30 after previous implant failure, cyst formation, localized bone necrosis & grafting	Implant removal at site #30 using a trephine bur followed by PRF grafting	Extraction & bone grafting associated with pre-existing periapical pathology involving the IAN canal
Time to symptom onset	Within 24 h after implant placement	Within 24 h after implant removal surgery	Immediately following extraction & grafting procedure
Presenting symptoms	Right-sided mandibular paresthesia involving lower lip, chin & posterior mandibular region	Right-sided mandibular paresthesia extending from the chin to the ipsilateral oral commissure	Persistent right-sided mandibular numbness involving the lower lip & chin
Clinical findings	Broad sensory deficit extending from the chin midline to the posterior ramus	Sensory deficit extending from the chin midline to the oral commissure	Well-demarcated area of paresthesia involving the lower lip & chin
Radiographic findings	CBCT showed implant positioned around 2 mm from the IAN canal without direct canal violation	CBCT showed osteotomy site nearly 2 mm from the IAN canal without direct canal penetration	Panoramic & periapical imaging showed tooth-related pathology extending into the IAN canal prior to extraction
Diagnostic impression	Indirect IAN injury secondary to implant proximity, compression, edema, or inflammatory insult	Indirect/compression-related IAN injury associated with implant removal osteotomy	IAN injury related to direct periapical involvement & probable canal violation during extraction
Treatment protocol	Weekly localized submucosal injections of ARA-290 (4 mg), TB-500 (10 mg), BPC-157 (1 mg), dexamethasone (8 mg) & bupivacaine 0.5% with epinephrine	Weekly localized submucosal injections of ARA-290 (4 mg), TB-500 (10 mg), BPC-157 (1 mg), dexamethasone (8 mg) & bupivacaine 0.5% with epinephrine	Weekly localized submucosal injections of ARA-290 (4 mg), TB-500 (10 mg), BPC-157 (1 mg), dexamethasone (8 mg) & bupivacaine 0.5% with epinephrine
Treatment duration	4 weeks	4 weeks	4 weeks
Follow-up findings	Progressive improvement throughout treatment	Progressive improvement throughout treatment	Progressive improvement throughout treatment & subsequent osseous healing
Final neurosensory outcome	Near-complete resolution with residual focal hypoesthesia (∼3 mm) inferior to the lower lip	Near-complete resolution with residual diminished sensation (∼2 mm) at the lateral chin	Nearly 95% reduction in subjective numbness with near-complete sensory recovery
Additional clinical outcome	Resolution of extensive paresthetic region	Resolution of extensive paresthetic region	Successful implant placement after 6 months of healing without complications
Adverse events	None reported	None reported	None reported

The biological reaction to peripheral nerve injury is highly intricate and comprises a synchronized sequence of cellular and molecular processes, such as Wallerian degeneration, Schwann cell (SC) stimulation, macrophage-dependent debris removal, vascularization, extracellular matrix remodeling, and axonal regeneration (5–7). Successful recovery relies not only on the inherent regenerative capability of peripheral nerves but also on the reestablishment of a permissive niche facilitating neural healing (8). In the mandibular canal, nonetheless, regeneration may be impaired by distinct anatomical limitations. Edema, vascular inadequacy, inflammation, and fibrosis can compromise axonal regrowth and contribute to persistent neurosensory disturbance (9, 10). Resultantly, functional recovery after IAN injury is mostly unpredictable, underscoring the necessity for therapeutic strategies that actively facilitate regeneration instead of merely attenuating symptoms (11, 12).

Contemporary management approaches for IAN injury remain mainly supportive. Corticosteroids are mostly used during the acute phase to alleviate edema and inflammation, while microsurgical interventions, pharmacologic neuroregulators, and local anesthetics are administered in some cases (13, 14). Although these strategies may mitigate secondary injury or enhance symptom control, they do not directly address the multifactorial biological signaling regulating nerve regeneration (15, 16). Consequently, treatment outcomes remain variable, and no universally accepted regenerative treatment has emerged for daily clinical application (3, 16).

Recently, regenerative peptides have garnered attention as promising biologic regulators of tissue healing and neural regeneration. Contrary to traditional treatments that target isolated components of the injury pathway, numerous peptide-mediated agents demonstrate pleiotropic activities that affect several regenerative cascades concurrently (17–19). Preclinical studies suggest that Body Protection Compound-157 (BPC-157) may enhance cytoprotection, endothelial stability, and angiogenesis, while regulating inflammatory pathways (20–22). Similarly, TB500 has been associated with enhanced cellular migration, cytoskeletal remodeling, and tissue healing in experimental models (23–25). ARA-290 has shown tissue-protective and immunoregulatory features in preclinical and selected early clinical studies (26–28). Nonetheless, these mechanisms have not been directly shown in patients with IAN injury and should be regarded as biologically plausible hypotheses instead of establishing clinical mechanisms.

While preclinical studies have shown promising impacts of vascularization, nerve regeneration, and functional recovery (17, 19, 29, 30), clinical evidence remains restricted, especially within oral and maxillofacial surgery. The application of these outcomes to IAN injury has yet to be systematically investigated, and reports elucidating their utility in dental nerve injuries are limited. Hence, this report presents three clinical cases of IAN injury managed with a multimodal peptide-mediated approach comprising peptide agents, adjunctive dexamethasone, and local anesthesia, and discusses the biological rationale underlying this strategy. These cases are presented solely as hypothesis-generating clinical observations intended to show the biological rationale for future investigation rather than to exhibit therapeutic efficacy.

## 2 Case 1

### 2.1 Patient information

A 38-year-old female with a prior history of Le Fort orthognathic surgery presented with the failure of a dental implant at site #30, which was related to cyst formation, localized bone necrosis, and abscess formation. The failed implant was removed, and the site underwent grafting, followed by an uneventful healing duration of around 8 months before reimplantation. Within 24 h post-implant placement, the patient reported the emergence of right-sided mandibular paresthesia. No significant systemic comorbidities were documented.

### 2.2 Clinical findings

Clinical assessment showed a substantial neurosensory deficit affecting the distribution of the right IAN, extending from the midline of the chin to the posterior ramus region (Figure 1C). The patient described modified sensation consistent with paresthesia in the lower lip and chin area.

**Figure 1:**
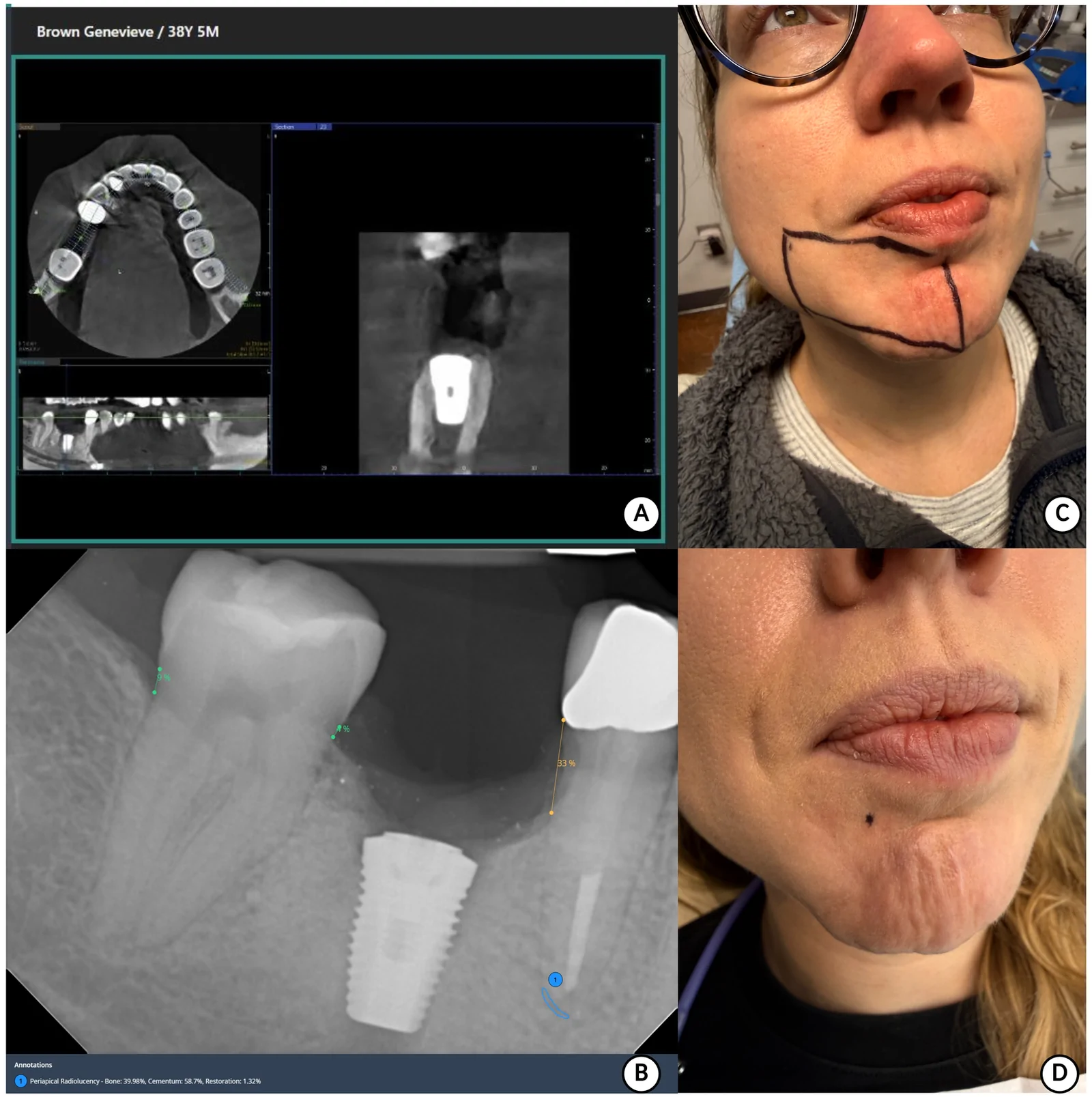
**(A)** CBCT multiplanar reconstruction (axial, cross-sectional, and panoramic views) showing implant placement at site #30 in proximity to the IAN canal, without clear radiographic evidence of direct canal violation. **(B)** Periapical radiograph showing the spatial association between the implant fixture and adjacent mandibular structures, with the implant positioned near the IAN canal but without encroachment. **(C)** Clinical picture showing the initial distribution of neurosensory deficit after IAN injury, with the affected region outlined on the right mandibular soft tissues, extending from the lower lip to the chin and lateral mandibular border. **(D)** Post-treatment clinical picture after completion of multimodal therapy, demonstrating marked resolution of the previously affected area, with only a small residual focal region of hypoesthesia inferior to the lower lip.

### 2.3 Diagnostic assessment

CBCT and periapical radiographic assessment revealed that the implant was placed in proximity to the IAN canal (nearly 2 mm), without any indication of direct canal violation or penetration (Figures 1A,B). The clinical findings were consistent with an indirect IAN injury, possibly associated with compression, edema, or local inflammatory responses instead of a direct nerve transection.

### 2.4 Therapeutic intervention

A multimodal treatment protocol comprising peptide agents (ARA-290 [4 mg], TB-500 [10 mg], and BPC-157 [1 mg]) together with dexamethasone (8 mg) and Bupivacaine 0.5% with 1:200,000 epinephrine (3.6 mL) weekly intraoral submucosal infiltrations was implemented over 4 weeks. Injections were precisely targeted to the affected sensory distribution.

### 2.5 Follow-up and outcomes

During follow-up, the patient reported gradual enhancement in neurosensory symptoms. At the 4-week follow-up, the previously documented area of paresthesia had substantially decreased, with only a minor residual region of hypoesthesia (almost 3 mm) persisting inferior to the vermilion border of the lower lip (Figure 1D). No treatment-associated adverse events were documented. Given the observational nature of this report and the absence of a control group, it cannot be determined whether this recovery was attributable to the treatment or reflected the natural course of nerve healing.

## 3 Case 2

### 3.1 Patient information

An 85-year-old patient presented with failure of a dental implant at site #30. The implant was extracted utilizing a trephine bur, and the osteotomy site was grafted with platelet-rich fibrin. Within 24 h post-surgery, the patient reported right-sided mandibular paresthesia.

### 3.2 Clinical findings

Clinical evaluation showed a sensory deficit extending from the midline of the chin to the ipsilateral oral commissure (Figure 2D). The extent and distribution of altered sensation were consistent with involvement of the IAN.

**Figure 2:**
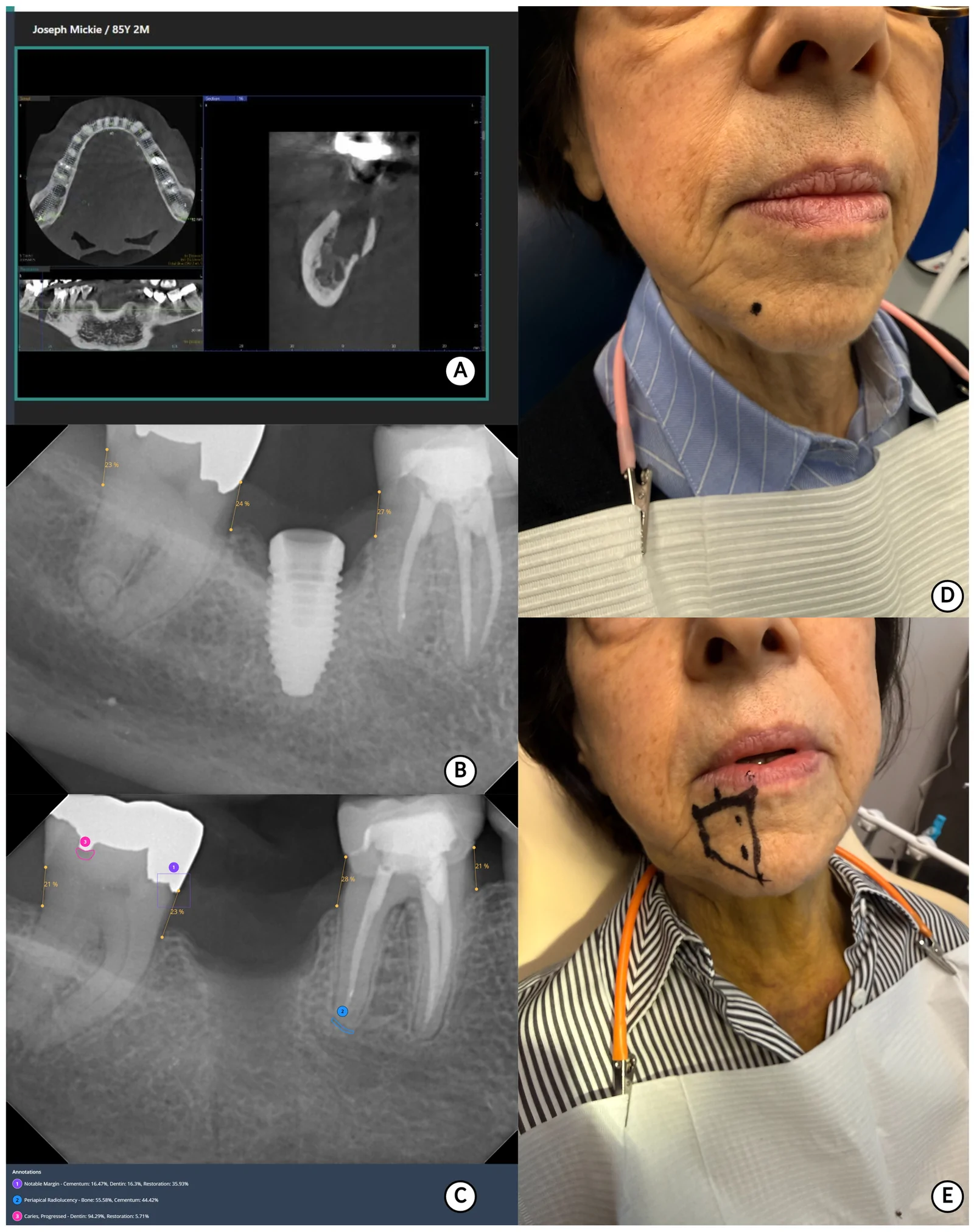
**(A)** CBCT multiplanar reconstruction (axial, cross-sectional, and panoramic views) showing the mandibular anatomy and the spatial course of the IAN canal in relation to the osteotomy site following implant removal at site #30, without clear evidence of direct canal violation. **(B)** Periapical radiograph showing the implant site adjacent to endodontically treated teeth, underscoring the proximity of the osteotomy site to the IAN canal and the potential for indirect or compression-associated nerve injury. **(C)** Follow-up peri-apical radiograph with annotated findings showing the relationship between the surgical site, adjacent dentition, and the IAN canal, emphasizing persistent proximity and associated risk to the nerve despite absence of overt radiographic penetration. **(D)** Clinical picture showing the initial distribution of neurosensory deficit after IAN injury, with the affected area outlined on the right lower lip and chin, reflecting localized paresthesia consistent with nerve involvement. **(E)** Post-treatment clinical picture after completion of multimodal intervention, exhibiting significant resolution of the previously affected area, with only a small residual focal region of diminished sensation of the chin.

### 3.3 Diagnostic assessment

CBCT imaging showed that the osteotomy site was in proximity to the IAN canal (around 2 mm), with no indication of direct canal penetration (Figures 2A–C). Periapical radiographs corroborated the close anatomical relationship between the surgical site and the mandibular canal. The observations indicated a compression-associated or indirect IAN injury.

### 3.4 Therapeutic intervention

The patient received weekly localized intraoral submucosal infiltrations over a period of 4 weeks, utilizing the same multimodal protocol consisting of peptide agents (ARA-290 [4 mg], TB-500 [10 mg], and BPC-157 [1 mg]) together with dexamethasone (8 mg) and Bupivacaine 0.5% with 1:200,000 epinephrine (3.6 mL).

### 3.5 Follow-up and outcomes

The patient reported gradual enhancement in sensory symptoms during follow-up. Upon completion of the therapy, the paresthetic region had significantly diminished in size. Only a minor residual area of diminished sensation (nearly 2 mm) persisted at the lateral aspect of the chin (Figure 2E). No complications related to treatment were reported. Nonetheless, since spontaneous recovery is common after certain IAN injuries, the relative contribution of the intervention cannot be determined.

## 4 Case 3

### 4.1 Patient information

A 48-year-old female presented after extraction of a tooth and subsequent bone grafting in the right posterior mandible. Postoperatively, she reported enduring numbness on the right side of the mandible, affecting the lower lip and chin (Figure 3B).

**Figure 3:**
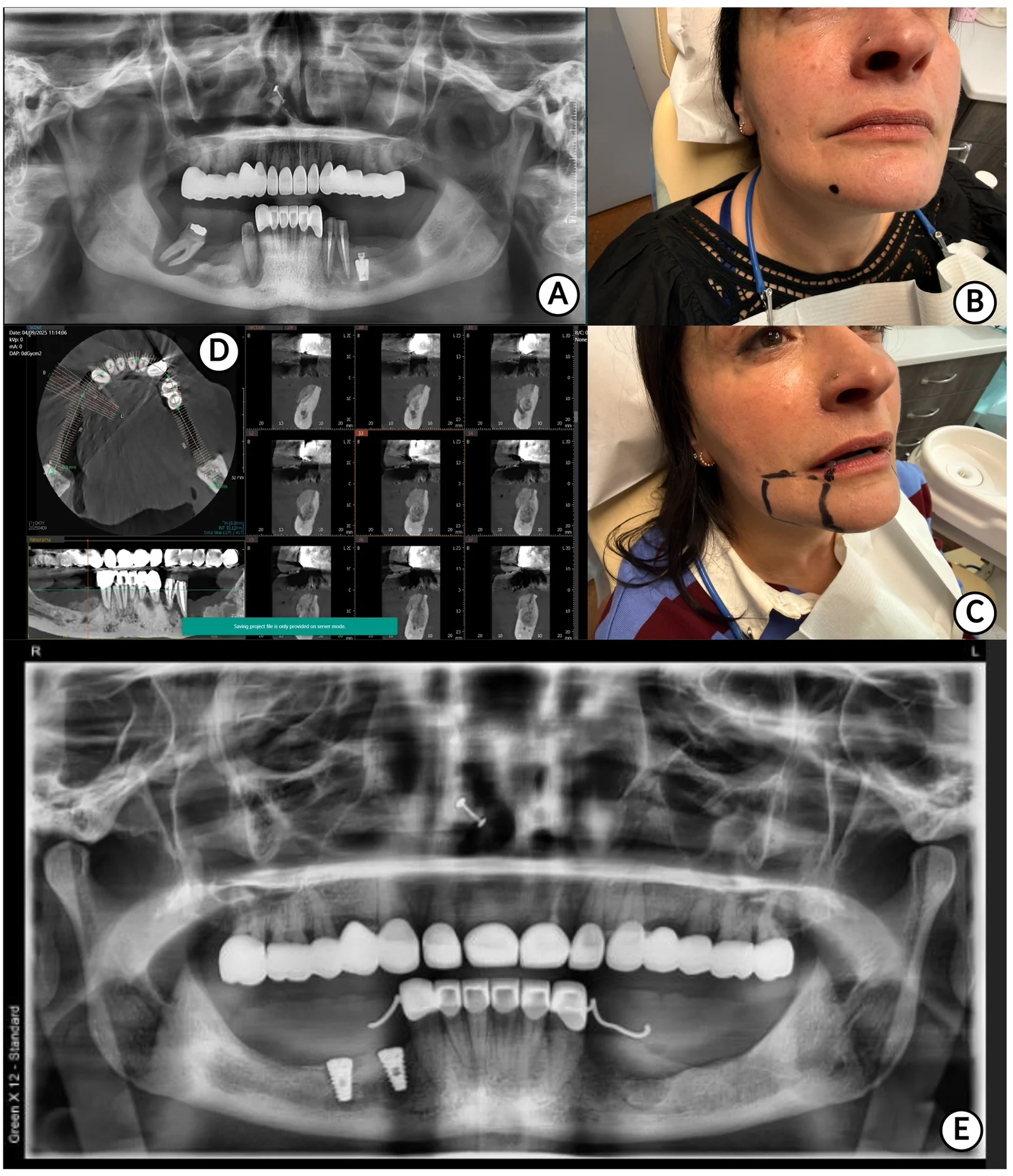
**(A)** panoramic radiograph showing the mandibular dentition and pre-extraction site in the right posterior mandible with tooth-associated lesions directly invading the IAN canal. **(B)** CBCT multiplanar reconstruction (axial, cross-sectional, and panoramic views) showing healing of the osseous defects 7 months after initiation of peptide therapy. **(C)** Panoramic radiograph showing dental implants placed into the healed alveolar jaw. **(D)** Clinical picture showing the initial distribution of neurosensory deficit on the right lower lip and chin, with the affected area outlined, consistent with IAN involvement post tooth extraction. **(E)** Post-peptide treatment clinical picture showing marked resolution of the previously affected region, with minimal residual focal hypoesthesia following peptide-based therapy.

### 4.2 Clinical findings

Clinical evaluation showed a clearly defined area of paresthesia in the right lower lip and chin, aligning with the distribution of the IAN.

### 4.3 Diagnostic assessment

Periapical and panoramic radiographs showed tooth-related pathology directly associated with the IAN canal before extraction. After extraction, the enduring neurosensory deficit indicated IAN injury (Figure 3A). Subsequent CBCT imaging revealed gradual osseous healing of the surgical site during follow-up.

### 4.4 Therapeutic intervention

The patient received weekly localized intraoral submucosal infiltrations over a duration of 4 consecutive weeks, following the same multimodal treatment approach described in Cases 1 and 2. Each treatment session comprised ARA-290 (4 mg), TB-500 (10 mg), BPC-157 (1 mg), dexamethasone (8 mg), and Bupivacaine 0.5% with 1:200,000 epinephrine (3.6 mL). The injections were administered into the affected sensory distribution of the IAN using the same infiltration technique used in the preceding cases.

### 4.5 Follow-up and outcomes

The patient reported progressive healing of sensation throughout the treatment course. By the conclusion of treatment, the subjective numbness had diminished by nearly 95%, with almost complete resolution of the affected region (Figure 3C). A six-month follow-up revealed satisfactory hard tissue repair (Figure 3D). Functional recovery was adequate to enable the placement of dental implants in the previously affected area without complications (Figure 3E), enabling implant placement in the previously affected area without complications. While recovery occurred during the intervention duration, this uncontrolled observation does not establish a causal association between the peptide protocol and the observed neurosensory enhancement. Table 1 shows the CARE case summary of three patients with IAN injury managed with a multimodal peptide-based regenerative protocol.

## 5 Discussion

IAN injury represents a challenging complication in oral and maxillofacial surgery owing to its unpredictable recovery trajectory and significant effect on patient quality of life. While numerous neurosensory dysfunctions may resolve without intervention, persistent dysesthesia, hypoesthesia, and paresthesia remain clinically relevant consequences after third-molar surgery, implant placement/removal, and other dentoalveolar interventions (1–4). This report describes three patients with postoperative IAN dysfunction who experienced gradual neurosensory recovery during follow-up after receiving a multimodal peptide-mediated intervention. Since this was an uncontrolled observational case series, these outcomes should not be interpreted as evidence of intervention efficacy. Spontaneous recovery, placebo effects, regression to the mean, variability in injury severity, and the simultaneous use of dexamethasone and local anesthetic all represent plausible alternative explanations for the observed clinical course. However, these cases offer preliminary clinical observations that, when considered together with existing mechanistic and preclinical evidence, justify further research in rigorously designed controlled studies.

An important finding across all three cases was the anatomical proximity between the injury site and the mandibular canal, without definitive radiographic indications of complete nerve transection in two of the patients. In Cases 1 and 2, CBCT imaging showed surgical sites situated nearly 2 mm from the IAN canal, indicating that indirect mechanisms, including inflammatory injury, compression, ischemia, and edema, may have played a more significant role than direct mechanical injury. Conversely, Case 3 involved pathology that extended into the IAN canal before extraction, reflecting a potentially more severe injury pattern. Despite the variance in etiology, all three patients showed a substantial decrease in sensory dysfunction after intervention. This outcome corroborates current comprehension that secondary injury processes, such as inflammation, vascular compromise, oxidative stress, and fibrosis, are central to identifying prolonged neurological outcomes following peripheral nerve injury (5–12, 31). By regulating these downstream pathways, regenerative therapies have been hypothesized to affect biological processes implicated in recovery, although this was not assessed in this study.

The biological justification for the multimodal approaches utilized in these cases is anchored mainly in mechanistic evidence derived from experimental models of peripheral nerve injury. Earlier clinical investigations have suggested that regenerative peptides may affect multiple biological processes involved in nerve repair, such as inflammatory signaling, vascular remodeling, ECM organization, SC biology, and axonal regeneration (5–12, 31–37). Nevertheless, none of these mechanisms were directly assessed in the present patients. Hence, references to these pathways should be interpreted as hypotheses explaining the biological rationale for peptide intervention instead of evidence that these regenerative processes transpired clinically in the presented cases.

Experimental studies suggest that ARA-290 stimulates innate repair receptors and may confer tissue-protective and immunoregulatory impacts (26–28, 38). Contrary to traditional anti-inflammatory modalities that broadly inhibit immune responses, ARA-290 seems to enhance a transition toward a reparative immune phenotype while maintaining necessary regenerative activities (26–28). These impacts may be especially significant in IAN injury, where extended oxidative stress and inflammatory pathways contribute to secondary neural injury and protracted healing (39–47). Preclinical studies have associated BPC-157 with regulation of inflammatory signaling, endothelial stability, and angiogenic function (20–22, 48, 49). The reestablishment of microvascular activity may be particularly essential within the mandibular canal, wherein even slight vascular insufficiency or edema can substantially compromise the vasa nervorum perfusion (9, 10, 50, 51). TB-500 has been associated in experimental studies with improved cellular migration, cytoskeletal remodeling, and tissue organization (23–25, 50, 51). These observations have resulted in the hypothesis that it may support SC-dependent repair and axonal regeneration; nonetheless, such pathways remain speculative in the context of the current clinical observations since no direct biological evaluations were conducted.

A vital component of the approach was its incorporation into traditional interventions. Bupivacaine and dexamethasone were used as adjuncts instead of primary regenerative treatments. Corticosteroids have historically been used to attenuate inflammation and edema during the acute phase of nerve damage, hence potentially restricting secondary neural injury (13, 14, 52–56). Similarly, long-term nociceptive inhibition using local anesthetics may mitigate neurogenic inflammation and the predisposition to central sensitization (57–60). Conceptually, the strategy hence amalgamated acute injury stabilization with biological rationale for tissue repair, aligning with modern paradigms of peripheral nerve recovery that focus on the significance of matching treatments to the evolving biological phases of damage and healing (61, 62).

IAN injury should be considered within the wider notion of the nerve-bone axis instead of an isolated peripheral nerve lesion (63). Since the IAN courses through the mandibular canal, ECM, immune cells, vasculature, bone, and neural tissue create an integrated neuro-skeletal niche in which inflammation, vascular compromise, fibrosis, and bone remodeling may collectively affect neurosensory recovery (63). This notion is especially significant in dentoalveolar surgery, where bone and adjacent neural structures are mostly injured concurrently. While these associations were not directly assessed in the present study, the nerve-bone axis offers a valuable biological system for comprehending the multifactorial nature of IAN injury and facilitates future research integrating neurosensory, radiographic, vascular, and bone remodeling outcomes.

Despite the promising findings, numerous alternative interpretations must be considered. Spontaneous recovery is a well-established phenomenon after minor IAN injury, especially in instances of limited axonotmesis and neuropraxia (11, 14, 15). Hence, it remains indeterminate to what extent the documented recovery was due to intervention instead of the inherent healing trajectory. Additionally, the multimodal essence of the approach impedes the detection of the contribution of any singular element. The observable advantages may stem from one agent, a subset of agents, or associations among several treatments. The likelihood of observer bias and placebo effects must also be recognized, especially since outcome evaluation depended mainly on clinical assessment and patient-documented sensory enhancements.

Hence, the current outcomes need to be regarded as hypothesis-generating instead of confirmatory in nature. However, they contribute to an emerging body of literature indicating that regenerative intervention may have a part in peripheral nerve recovery. Existing literature facilitating peptide-mediated therapies remains primarily preclinical and is concentrated on models involving diabetic neuropathy, sciatic nerve injury, and non-craniofacial nerve platforms (17, 19, 63–66). In oral and maxillofacial surgery, clinical data remains scarce. Resultantly, these cases offer preliminary descriptive clinical observations that are directly pertinent to dental and implant-associated nerve injuries.

Many limitations require recognition. Limited sample size, absence of a control group, and treatment allocation were not randomized. An additional limitation is the absence of standardized quantitative neurosensory evaluation. Recovery was mainly assessed using routine clinical examination, patient-reported changes in sensation, and serial photographic documentation. Objective examinations, such as validated patient-reported outcome measures, visual analog pain scores, electrophysiological studies, quantitative sensory testing, thermal discrimination, pin-prick testing, two-point discrimination, and static light-touch testing, were not obtained prospectively. Hence, the magnitude of sensory recovery cannot be objectively quantified or directly compared with other published studies using standardized neurosensory outcome measures. Another important limitation is the inability to isolate the effects of the peptide components from those of adjunctive therapy. All three patients received an identical multimodal protocol containing ARA-290, TB-500, BPC-157, dexamethasone, bupivacaine, and epinephrine. Hence, it is impossible to determine whether the observed clinical course resulted from one agent, a combination of agents, associations among interventions, or the expected natural history of recovery. Moreover, follow-up duration was inconsistent, restricting evaluation of long-term stability. Furthermore, the long-term safety profiles, pharmacokinetics, optimal dosing approaches, and regulatory status of many peptide-mediated agents remain inadequately characterized. Moreover, since no untreated comparison group was available, it was not possible to differentiate intervention-associated effects from the expected natural history of recovery after peripheral nerve injury. Hence, this study should be interpreted as a descriptive observational case series designed to generate hypotheses rather than assess therapeutic efficacy.

Future investigations should emphasize prospective controlled trials assessing peptide-mediated regenerative approaches in patients with well-characterized IAN injuries. Longer follow-ups, imaging-dependent evaluation, quantitative sensory assessments, and standardized neurosensory outcome measures will be required to identify whether these interventions offer a significant advantage beyond traditional management and spontaneous healing. Finally, comparative studies assessing isolated versus combination peptide interventions may facilitate clarifying potential synergistic impacts and determining optimal therapeutic strategies.

## 6 Conclusion

The present study describes three patients with IAN injury who experienced neurosensory recovery during follow-up after receiving a multimodal peptide-mediated protocol. Because of the uncontrolled observational design, lack of randomization, blinding, standardized neurosensory evaluation, and untreated controls, these findings should not be interpreted as evidence of treatment efficacy or causality. Rather, these cases represent hypothesis-generating clinical observations that support further prospective controlled trials assessing the biological rationale and potential role of regenerative peptide-mediated strategies in the management of IAN injury.

## Data Availability

The original contributions presented in the study are included in the article/Supplementary Material, further inquiries can be directed to the corresponding author.
